# Does Holistic Processing Require a Large Brain? Insights From Honeybees and Wasps in Fine Visual Recognition Tasks

**DOI:** 10.3389/fpsyg.2018.01313

**Published:** 2018-07-31

**Authors:** Aurore Avarguès-Weber, Daniele d’Amaro, Marita Metzler, Valerie Finke, David Baracchi, Adrian G. Dyer

**Affiliations:** ^1^Centre de Recherches sur la Cognition Animale, Centre de Biologie Intégrative (CBI), Université de Toulouse, CNRS, UPS, Toulouse, France; ^2^Institut für Zoologie III (Neurobiologie), Johannes Gutenberg Universität Mainz, Mainz, Germany; ^3^Department of Anatomy II, University of Cologne, Cologne, Germany; ^4^School of Media and Communication, Royal Melbourne Institute of Technology, Melbourne, VIC, Australia; ^5^Department of Physiology, Monash University, Clayton, VIC, Australia

**Keywords:** *Apis mellifera*, configural processing, face recognition, hierarchical stimuli, holistic processing, hymenopterans, *Vespula vulgaris*, visual cognition

## Abstract

The expertise of humans for recognizing faces is largely based on holistic processing mechanism, a sophisticated cognitive process that develops with visual experience. The various visual features of a face are thus glued together and treated by the brain as a unique stimulus, facilitating robust recognition. Holistic processing is known to facilitate fine discrimination of highly similar visual stimuli, and involves specialized brain areas in humans and other primates. Although holistic processing is most typically employed with face stimuli, subjects can also learn to apply similar image analysis mechanisms when gaining expertise in discriminating novel visual objects, like becoming experts in recognizing birds or cars. Here, we ask if holistic processing with expertise might be a mechanism employed by the comparatively miniature brains of insects. We thus test whether honeybees (*Apis mellifera*) and/or wasps (*Vespula vulgaris*) can use holistic-like processing with experience to recognize images of human faces, or Navon-like parameterized-stimuli. These insect species are excellent visual learners and have previously shown ability to discriminate human face stimuli using configural type processing. Freely flying bees and wasps were consequently confronted with classical tests for holistic processing, the part-whole effect and the composite-face effect. Both species could learn similar faces from a standard face recognition test used for humans, and their performance in transfer tests was consistent with holistic processing as defined for studies on humans. Tests with parameterized stimuli also revealed a capacity of honeybees, but not wasps, to process complex visual information in a holistic way, suggesting that such sophisticated visual processing may be far more spread within the animal kingdom than previously thought, although may depend on ecological constraints.

## Introduction

Humans and other primates have a remarkable ability to detect and visually identify conspecifics on the basis of their faces, which is a crucial capacity in our social interactions (Kanwisher et al., 1997; Pascalis et al., 2002; Wilmer et al., 2010; Young and Burton, 2017). A key mechanism of human face processing is that the visual system does not only use salient elemental features like hair, eyes, nose, or mouth to enable recognition, but it is rather the relationships between features or the configuration of a face that potentially allows for the seemingly advanced ability of humans to recognize conspecific faces (Carey and Diamond, 1977; Tanaka and Sengco, 1997; Collishaw and Hole, 2000; Maurer et al., 2002; Peterson and Rhodes, 2003).

Relationship processing between elemental features, a cognitive ability known as configural processing in visual cognition field, is considered to improve visual recognition accuracy. Three plausible levels of configural processing for face stimuli have been defined based upon human psychophysics experiments and/or neurophysiological recordings (Maurer et al., 2002). These three levels include (i) sensitivity to first-order relations where the spatial relationships between elemental features are processed (e.g., detecting a face because its features comprise a uniformed arrangement in which eyes are located above the nose which is located above a mouth); (ii) holistic processing, in which elemental features are bound together into a gestalt, and (iii) sensitivity to second-order relationships, in which slight variations of distances between features are perceived. Access to the first level of proposed processing is evidenced for example by a capacity to detect faces amongst considerable background noise like inverted two-tone Mooney faces (Maurer et al., 2002) and allow us to categorize stimuli as faces therefore activating specialized brain areas and specific holistic processing (Kanwisher, 2000; Maurer et al., 2002). Experimental access to holistic processing is achieved using stimuli manipulations including the part-whole effect and the composite-face effect (Carey and Diamond, 1977; Tanaka and Sengco, 1997; Collishaw and Hole, 2000; Maurer et al., 2002; Peterson and Rhodes, 2003). Indeed, because upright faces engage holistic processing, it is difficult to extract individual feature information separately. Thus, it is harder to recognize part of a face (e.g., the eyes) when perceived in isolation while the performance is restored when these features are replaced in the context of the full face (Part-Whole effect). Additionally, the creation of a composite face with features from different faces disrupts feature recognition as the composite face is processed holistically as a novel face (Composite face effect) (Carey and Diamond, 1977; Tanaka and Sengco, 1997; Collishaw and Hole, 2000; Maurer et al., 2002; Peterson and Rhodes, 2003). It is then often assumed that holistic representations enable second-order relationship processing that promotes reliable recognition among highly similar faces (Farah et al., 1998; Maurer et al., 2002; McKone et al., 2007; Taubert et al., 2011). Interestingly, it has also been suggested that holistic processing may operate as a general mechanism to aid reliable recognition from other competing objects in a complex visual environment (Tanaka and Gauthier, 1997; Farah et al., 1998; McKone et al., 2007; Taubert et al., 2011). Indeed, whilst the human and primate brain does have dedicated neural circuitry involved in face processing like the fusiform face area (Kanwisher et al., 1997; Kanwisher, 2000; Tsao et al., 2006), such areas do also facilitate recognition of other non-face stimuli when subjects are experts (Gauthier and Tarr, 1997; Gauthier et al., 2000).

Recently, the question on whether animals with different neural architecture may be able to process faces has received increased interest. There is growing evidence that animals including non-human primates (Sugita, 2008; Parr, 2011), dogs (Huber et al., 2013), sheep (Kendrick et al., 2001; Morton et al., 2018), magpies (Lee et al., 2011), house sparrows (Vincze et al., 2015), or fish species (Levey et al., 2009; Siebeck et al., 2010; Newport et al., 2016; Wang and Takeuchi, 2017) can reliably process images of human faces despite having very different neural architectures, and in many cases no shared evolutionary history to enable experience at viewing human faces [see Leopold and Rhodes (2010) for a review]. However, only a few studies studied the existence of holistic processing of conspecific or human faces in animals (Burke and Sulikowski, 2013). In parallel, the question of configural/holistic processing for other visual objects has been mainly investigated by using Navon-like hierarchical stimuli (stimuli showing a global shape or configuration created by the spatial arrangement of local shapes). Most tested species demonstrated a preference to process local information rather than the global configuration [e.g., baboons (Fagot and Deruelle, 1997), capuchin monkeys (Truppa et al., 2017), or chicks (Chiandetti et al., 2014)]. To date, only Humans (Navon, 1977), a fish species *Xenotoca eiseni* (Truppa et al., 2010) and honeybees (Avarguès-Weber et al., 2015) showed consistent global preference suggesting a general importance of visual configural processing in these species.

In this context, some social insects species became promising models of visual configural processing due to experimental access combined with evidence of impressive visual recognition abilities including face processing of conspecifics (Tibbetts, 2002; Sheehan and Tibbetts, 2011), human faces (Dyer et al., 2005; Dyer and Vuong, 2008; Avarguès-Weber et al., 2017), or configural processing of parameterised visual stimuli (Avarguès-Weber et al., 2010b, 2015; Howard et al., 2017). Thus, a paper wasp species (*Polistes fuscatus*) was shown capable of individual recognition of conspecifics (Tibbetts, 2002). In a follow-up study (Sheehan and Tibbetts, 2011), the recognition ability of *P. fuscatus* foundresses was evaluated for visual stimuli including conspecific faces, prey items, complex geometric shapes, or conspecific faces where configuration had been manipulated. *P. fuscatus* wasps’ recognition level for conspecific faces was superior to all other stimuli in particular faces with altered configuration (Sheehan and Tibbetts, 2011). This evidence from *P. fuscatus* wasps shows that individual recognition via subtle visual discrimination is also possible in insects with potential convergence of visual strategies based on configural processing with mammals (Avarguès-Weber, 2012; Chittka and Dyer, 2012). Further works on wasps suggest that face recognition may have evolved several times in insects depending upon ecological constraints (Baracchi et al., 2015, 2016).

The fact that paper wasps could recognize conspecifics (Tibbetts, 2002) also lead to research testing whether honeybees might be able to recognize human faces (Dyer et al., 2005). When trained in an appetitive-aversive differential conditioning protocol to discriminate pictures of human faces chosen from a standard face recognition test as difficult to differentiate for human subjects (Warrington, 1996), free-flying honeybees could reliably recognize the rewarded target face even in the presence of very similar and novel distractor faces (Dyer et al., 2005). Subsequent work showed that honeybees could interpolate information from multiple viewpoints of faces to enable face recognition at novel viewpoint angles (Dyer and Vuong, 2008), or use configural mechanisms to enable first order processing of face stimuli (Avarguès-Weber et al., 2010b). Finally, in a recent experiment free flying wasps *Vespula vulgaris* were shown also capable to learn the same human faces pictures with performance similar to that of honeybees (Avarguès-Weber et al., 2017).

In the current study, we employ the framework for configural face processing proposed by Maurer et al. (2002) to test the capacity of both the honeybee (*Apis mellifera*) and the wasp (*V. vulgaris*) to process greyscale pictures of human faces used in previous studies (Dyer et al., 2005; Avarguès-Weber et al., 2017) as well as Navon-like geometrical hierarchical stimuli using a holistic processing mechanism. These visual objects, classically used in visual cognition studies, were chosen because of their complexity offering better chance to require configural processing to resolve them. In addition, the high perceptual difference between both types of pictures allows investigating whether holistic processing could be a general mechanism. Both of these insect species are visually active foragers, but neither has any evolutionary history of using visual information for recognition of human faces. We employ adaptations of the part-whole effect, and the composite-face effect experiments typically used to evaluate face processing in humans. Importantly, our study does not directly attempt to make inferential analyses between insect and human species, but seeks to understand whether our test model species show evidence of holistic-like processing in an attempt to gain insights into whether holistic processing is a mechanism that is general to visual systems in nature for fine discrimination.

## Materials and Methods

### Experiment 1: Human Faces Pictures

**FIGURE 1:**
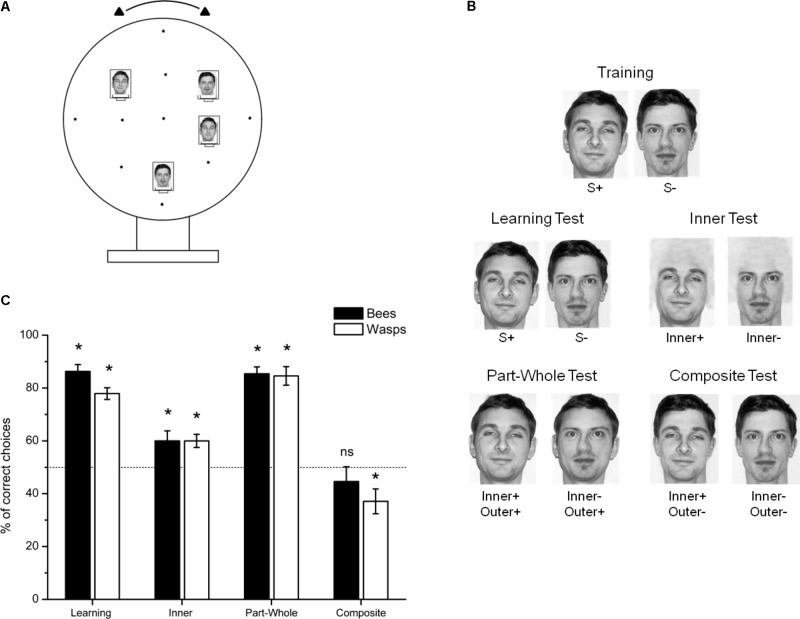
Experiment with human face pictures. **(A)** Schematic representation of the experimental setup. **(B)** Stimuli used for training and the non-reinforced tests. **(C)** Mean ± SEM percentage of choices for the correct stimulus on the 20 total test choices in each of the non-reinforced tests. The black bars show honeybees results (*N* = 12) while the white bars represent the wasps results (*N* = 12). The dashed line indicates chance level (^∗^*p* < 0.05). The pictures are used and reproduced with permission from Psychology Press, the original publisher (Warrington, 1996).

Experiments were conducted in 2013 at Mainz University with individually tagged and tested honeybees (*A. mellifera* L.) and wasps (*V. vulgaris*) trained by providing sucrose rewards to freely visit the experimental apparatus, a 50 cm diameter vertical screen which could be rotated to vary the spatial arrangement of the stimuli presented on it (Dyer et al., 2005; Dyer and Vuong, 2008) (**Figure 1A**). Only one individual was present at a time at the apparatus during the training and the tests. Two achromatic human faces from a standard face recognition test (Warrington, 1996) and used previously to investigate human face recognition abilities in bees (Dyer et al., 2005) and wasps (Avarguès-Weber et al., 2017) were chosen as complex visual stimuli to be discriminate. Four stimuli (two identical S+ and two identical S− stimuli; **Figures 1A,B**) were presented simultaneously on top of landing platforms offering a 10 μL drop of either a 25% (vol/vol) sucrose solution (S+) or a 60 mM quinine hemisulfate solution (S−), which promotes enhanced visual discrimination performances (Avarguès-Weber et al., 2010a). The reinforcement contingency was balanced between tested subjects. The face stimuli were attached on freely rotating 6 cm × 8 cm hangers that could be positioned in a number of random spatial positions and rearranged during the training by a rotation of the whole screen or manual displacements of the hangers (**Figure 1A**).

Before returning to the nest to deliver the sucrose collected, the bees or wasps typically made four to six choices (landing on a stimulus platform). Training length was chosen after pilot experiments to assure both species obtained a high level (≈80% of correct choices) of discrimination between the training faces, and a capacity to identify the target when presented with the inner part only of the training faces (*Inner Test*, see description of the tests below) consistent with previous evidence reported in Avarguès-Weber et al. (2010b). We thus used a training length of 180 choices for each bee, and 90 choices were necessary to reach a similar level of performance with the wasps. However, an inferential interpretation of the effect of training length between species was not a goal of the current study. In particular, experiments with bees and wasps were not conducted in parallel and may therefore have been subjected to differential seasonal effects for example. In this regard, our pilot tests found wasps only reliably forage for sucrose solution in the last weeks of summer which induces very limited experimental opportunity to test this species in free-flying conditions. Stimuli and landing platforms were washed with ethanol between foraging bouts and before the tests.

After training was completed, three non-reinforced test conditions were presented to the bees and wasps in which the first 20 choices were recorded (**Figure 1B**). The different test sessions were intermingled by three refreshing foraging bouts with the training conditions to maintain motivation. First, a *Learning test* presenting the training stimuli allowed accessing S+/S− discrimination level after the training session (**Figure 1B**). We then analyzed as a control the capacity of bees and wasps to discriminate both training face stimuli when only the stimuli inner parts were available (*Inner test*; **Figure 1B**). The comparison of performance level between the *Inner test* and the *Part-Whole Test* in which the S+ face was presented against a composed face (S− inner part surrounding by S+ outer features) was used as an indicator of holistic processing in the tested animals (**Figure 1B**). Both the *Inner test* and the *Part-Whole test* could only be resolved by the discrimination of the S+ vs. S− inner parts. The only difference between either test is that the inner parts were replaced in the context of a full image in the *Part-Whole test*. Thus, if bees’ or wasps’ visual recognition systems are sensitive to the “part-whole” effect, performance of the *Part-Whole test* should be higher than performance of the *Inner test* in which inner stimuli features are presented in isolation.

Finally, the *Composite test* aimed to investigate a potential composite face effect by offering a choice between a composed stimulus (S+ inner part and the S− outer part) and the S− face stimulus. Performance in this test should be lower than in the *Inner test* if the tested subjects were relying on holistic processing to solve the discrimination task.

### Experiment 2: Hierarchical Navon-Like Parameterized Stimuli

**FIGURE 2:**
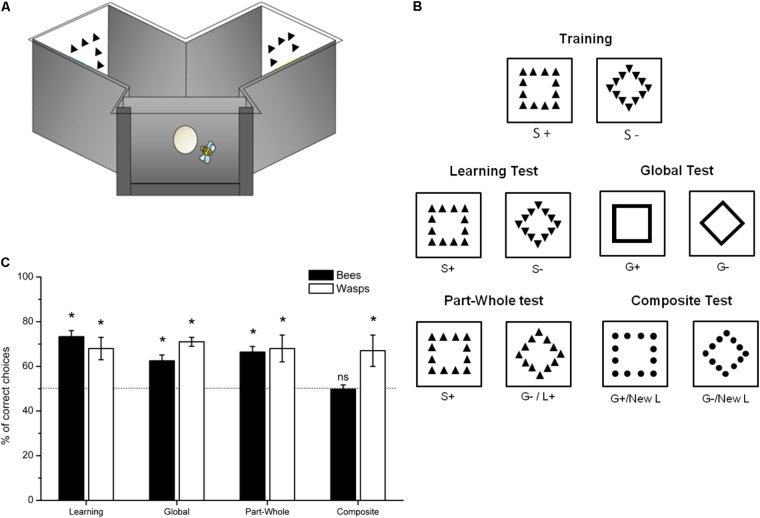
Experiment with Navon-like stimuli. **(A)** Schematic representation of the experimental setup. **(B)** Stimuli used for training and the non-reinforced tests. **(C)** Mean ± SEM percentage of choices for the correct stimulus on the 20 total test choices in each of the non-reinforced tests. The black bars show honeybees results (*N* = 10) while the white bars represent the wasps results (*N* = 6). The dashed line indicates chance level (^∗^*p* < 0.05).

This experiment was conducted with individually tagged and tested honeybees (in 2012, Mainz University) and wasps (in 2017, Mainz University) trained to freely visit a Y-maze setup covered by an ultraviolet transparent Plexiglas ceiling (**Figure 2A**). The entrance of the maze led to a decision chamber, where the flying insect could choose between the two arms of the maze (**Figure 2A**). One stimulus was presented vertically on each back wall of the arms which were placed at 15 cm from the decision chamber (**Figure 2A**). Such a setup allows for a controlled viewing distance as choices are recorded when the insect leaves the decision chamber thus entering one arm of the Y-maze. The visual angle subtended by the stimuli at the decision point was consequently controlled so that both small local features and large global features of the hierarchical stimuli were easily perceived by the animals.

The training phase consisted of a differential conditioning task with two hierarchical compound stimuli including a 11 cm square composed by the spatial arrangement of 12 repetitions of 1-cm up-triangles and a 11 cm diamond (45° rotated square) composed by 12 repetitions of 1-cm down-triangles (**Figure 2B**). For each tested subject, one of these stimuli was set in a balanced design as the S+ and associated with a 25% sucrose solution while the other was set as the S− and associated with a quinine solution (60 mM). Solutions were delivered in the center of each stimulus by means of transparent micropipettes. Between each foraging bout, the respective side of the S+ and the S− was allocated to the left or the right arm of the maze in a pseudo random fashion (e.g., the same stimulus was not presented in the same side more than twice in a row). If the subject chose the arm in which the S+ was presented, it could drink the sucrose solution *ab libitum* before returning to the nest. If the subject chose the S− arm, it was allowed to taste the quinine solution and then to fly back freely to the alternative arm where it could drink the sucrose solution; but only the first choice, recorded when the animal entered an arm, was counted. The training lasted 36 choices which correspond to 36 foraging bouts in this setup. This training length assured similar level of performance both for the bees and the wasps.

After training was completed, the subjects faced a *Learning test* with fresh S+ and S− stimuli (**Figure 2B**). Then four different non-reinforced transfer tests were proposed in a random sequence order intermingled by three refreshing training bouts (**Figure 2B**). During the tests, contacts with the surface of the stimuli were counted for 45 s.

As a control, global feature learning was assessed by analyzing the insects’ capacity to recognize the S+ global shape (square or diamond) vs. the S− global shape when presented in isolation, i.e., in the absence of the local features thus created by 1-cm wide plain lines (*Global test*; **Figure 2B**).

To evaluate the existence of the part-whole effect as indicator of holistic processing, we compared performance in the *Global test* to performance in the *Part-Whole test* offering a choice between the S+ global shape constructed by the S+ local elements (S+ stimulus) versus the S− global shape constructed also by the S+ local elements (composed stimulus S+/S−). In both tests, only the global information could be used as a cue but was presented in isolation in one case (*Global Test*) and in the whole context of a Navon-like stimulus in the other case (*Part-Whole Test*) (**Figure 2B**).

We then tested whether adding a novel local cue would impede recognition of the global cue (composite effect) in the *Composite test* (G+/Lnew vs. G-/Lnew) (**Figure 2B**). The performance in this test was also compared to the recognition level in the *Global test* where only global cues were available.

### Statistical Analysis

Performances during the tests (proportion of correct choices out of the 20 test choices; a single value by subject) were analyzed with a generalized linear model (GLM) selecting a binomial distribution and a logit link function. This model only included the intercept term to test for a significant difference between the mean proportion of observed correct choices (p) and the proportion of choices expected by chance (*p* = 0.5). The stimulus set as rewarded (categorical factor) never had a significant influence on the performance (*p* > 0.05) and data were, therefore, pooled for the tests analysis. The performances of the different tests were compared with a GLMM in which individuals were considered as a random factor to account for the repeated measurement design while the type of test was set as a categorical variable. The analyses were performed with R software, version 3.3.2 (R Development Core Team), lme4 package (Bates et al., 2014).

## Results

### Experiment 1: Human Faces Pictures

#### Honeybees

Honeybees (*N* = 12) succeeded in learning the discrimination task between the two human faces (S+ vs. S−; **Figure 1C**). The discrimination performance was significantly higher than chance level in the non-reinforced *Learning test* where the bees had to choose between the S+ and S− stimuli [*N* = 12; 86.3 ± 2.6 (mean ± SEM) % of correct choices; GLM: *z* = 9.80, *p* < 0.001; **Figure 1C**]. There was no significant influence of the face used as S+ stimulus (*z* = 0.19, *p* = 0.85).

The bees were still capable of recognizing the training stimuli when only the inner parts were available (*Inner test*: 60.0 ± 3.8% of correct choices; *z* = 3.08, *p* = 0.002; **Figure 1C**). However, performance was significantly lower than for the whole faces (*Inner test* versus *Learning test*: GLMM: *z* = 6.29, *p* < 0.001; **Figure 1C**).

In the *Part-Whole test*, adding the S+ outer part to re-create whole faces allowed the restoration of the *Learning test* performance level although the bees could only rely as in the *Inner test* on the inner parts to discriminate both stimuli. Indeed, the outer parts were identical for both options (85.5 ± 2.6% of correct choices; *z* = 9.67, *p* < 0.001; comparison with the *Learning test*: *z* = 0.26, *p* = 0.79 and with the *Inner test*: *z* = 6.09, *p* < 0.001; **Figure 1C**). The honeybees seem thus sensitive to the “part-whole” effect as recognition of a part of the training stimulus was facilitated when presented in the context of a whole face.

When confronted to the *Composite test* in which the distractor (S−) outer feature was added to the inner part of the S+ face, the bees failed to recognize such composite stimulus as being more similar to the S+ face than the full S− alternative option (44.6 ± 5.6% of correct choices; *z* = 1.66, *p* = 0.09; **Figure 1C**). Results from this test suggest that honeybees are sensitive to the “composite-face” effect as they had greater difficulty to recognize the S+ inner feature when placed in the context of an incorrect whole face than presented in isolation (*Composite test* versus *Inner test*: *z* = 3.40, *p* < 0.001; **Figure 1C**).

#### Wasps

The wasps (*N* = 12) trained to discriminate the S+ and S− human faces successfully learned the task after 90 reinforced choices (77.9 ± 2.2% of correct choices in the *Learning test*; *z* = 8.10, *p* < 0.001; **Figure 1C**) and were able to use only the inner features of the faces to recognize the S+ stimulus (*Inner test*: 60.0 ± 2.5% of correct choices; *z* = 3.08, *p* = 0.002; **Figure 1C**) although performance level was significantly lower than with the whole face (*Learning test* versus *Inner test*: *z* = 4.20, *p* < 0.001; **Figure 1C**). There was no significant influence of the face used as S+ stimulus (*z* = 0.25, *p* = 0.80).

The wasps also showed restored performance when full faces were presented in the *Part-Whole test* even if the available information to solve the discrimination task remained the inner features only as for the *Inner test* (84.6 ± 3.5% of correct choices, *z* = 9.52, *p* < 0.001; *Part-Whole test* versus *Learning test*: *z* = 1.86, *p* = 0.06; *Part-Whole test* versus *Inner test*: *z* = 5.84, *p* < 0.001; **Figure 1C**). The wasps seem consequently also sensitive to the “Part-Whole effect” when extensively trained with complex visual stimuli.

Finally, in the *Composite test*, the wasps not only failed to recognize the S+ inner features when surrounded by the S− outer features (“Composite-face effect”) but showed significant preference for the S− stimulus suggesting novelty aversion for the composed stimulus (37.1 ± 4.7% of correct choices; *z* = 3.96, *p* < 0.001; *Composite test* versus *Inner test*: *z* = 4.98, *p* < 0.001; **Figure 1C**). A similar tendency although not significant (44.6% of correct choices, *p* = 0.09; see above) was also observed in bees.

### Experiment 2: Hierarchical Navon-Like Parameterized Stimuli

#### Honeybees

Honeybees (*N* = 10) successfully learned to discriminate the S+ and S− hierarchical stimuli as performance in the *Learning test* was significantly above chance level (73.3 ± 2.7% of correct choices; *z* = 6.66, *p* < 0.001; **Figure 2C**). There was no significant influence of the stimulus used as S+ (*z* = 1.18, *p* = 0.24). The bees were capable to recognize the S+ global shape even when drawn with a solid line (interpolation) instead of distinct local features (*Global test*: 62.5 ± 2.6% of correct choices; *z* = 3.82, *p* < 0.001; **Figure 2C**) but this transformation resulted in poorer performance than in the *Learning test* (*z* = 2.43, *p* = 0.02; **Figure 2C**).

The bees behaved consistently with a sensitivity to the “part-whole effect” with parameterized stimuli as with the face stimuli: adding the same local features (L+) to the global shapes (*Part-Whole test*: G+L+ versus G-L+), thus re-constructing full hierarchical stimuli while still only offering the global information to allow solving the discrimination task, induced restored performance to a level similar to the *Learning test* performance (66.4 ± 2.5% of correct choices, *z* = 4.81, *p* < 0.001; *Part-Whole test* versus *Learning test*: *z* = 1.62, *p* = 0.11) although not significantly different from the *Global test* performance (*Part-Whole test* versus *Global test*: *z* = 0.82, *p* = 0.41; **Figure 2C**).

When facing the stimuli of the *Composite test* created by using novel local elements (dots), the bees failed to recognize the S+ and S− global features (49.7 ± 2.0% of correct choices, *z* = 0.20, *p* = 0.84; *Composite test* versus *Global test*: *z* = 2.85, *p* = 0.004; **Figure 2C**) thus suggesting again the influence of the “composite-face effect.”

#### Wasps

The wasps (*N* = 6) trained to discriminate S+ from S− hierarchical Navon-like stimuli successfully solved the task as shown by their performance in the Learning test (68.0 ± 5.1% of correct choices, *z* = 3.11, *p* = 0.002; **Figure 2C**). There was no significant influence of the stimulus used as S+ (*z* = 0.55, *p* = 0.58). They were also capable of interpolating the learnt stimuli to their global shape in the absence of local features (Global test: 71.3 ± 1.9% of correct choices; *z* = 2.52, *p* = 0.01; **Figure 2C**). Interestingly, removing local features did not impede wasps’ performance (*Global test* versus *Learning test*: *z* = 0.44, *p* = 0.66; **Figure 2C**). A similar level of performance was obtained when the hierarchical structure was restored by adding the S+ local features to both global information (*Part-Whole test*: 68.0 ± 5.8% of correct choices; *z* = 2.20, *p* = 0.03; *Part-Whole test* versus *Learning test*: *z* = 0.73, *p* = 0.47; **Figure 2C**). The wasps also did not appear to experience difficulty in recognizing the global information when novel local features were used (Composite test: 66.5 ± 6.6% of correct choices, *z* = 4.25, *p* < 0.001; *Composite test* versus *Learning test*: *z* = 1.22, *p* = 0.22; **Figure 2C**). Thus, in this particular experiment, the wasps did not seem to use holistic-like processing mechanism to recognize simplified parameterized stimuli, in contrast to our results with honeybees.

## Discussion

In this paper, we evaluated whether either of two hymenopteran species with relatively small brains of less than a million neurons might have a capacity for holistic processing of human faces, and parameterized stimuli, following the definitions for configural processing outlined by Maurer et al. (2002). Using the part-whole effect type experiment both honeybees and wasps showed a significant improvement to discriminate between inner features of the faces when they were shown together with identical outer features in a holistic stimulus than when presented in isolation (**Figure 1**). However, visual processing was totally disrupted when the correct face inner features were combined with the outer features of the distractor, showing that both bees and wasps were sensitive to the composite-face effect in their visual processing of stimuli (**Figure 1**). Thus, both bee and wasp species showed evidence consistent with holistic processing when having to recognize pictures of human faces, even though neither species has any ecological reason of having experience with human faces.

In the experiments with parameterized stimuli, honeybees also exhibited choice behavior consistent with holistic processing as performance was lower when bees had only access to the global features than when presented together with the local features (part-whole effect) and the bees’ choices collapsed to chance level when the same global features were shown together with novel local features (composite effect) (**Figure 2**). However, in wasps, no change in the capacity to recognize global features was observed, neither when presented in isolation, in a whole hierarchical context, nor together with novel local cues (**Figure 2**). Wasps did not seem consequently to rely on holistic-like processing with these particular stimuli. Different hymenopteran species thus process and implement various forms of configural processing in different ways. Interestingly, honeybees are known to process Navon stimuli with a global preference consistent with configural processing, but this preference could be modulated with priming to local stimulus elements (Avarguès-Weber et al., 2015), showing evidence of plasticity in the application of visual processing rules by honeybees. In bees, the sensitivity to some contextual visual illusions also considered as dependent on configural processing could also be modulated and is in particular under the influence of the conditioning procedure (Howard et al., 2017). The influence of testing procedure might also be at the origin of the difference in Global/Local processing between species as the fish species (Truppa et al., 2010) and bees (Avarguès-Weber et al., 2015) were the only animals tested while having the possibility to move toward the stimuli thus promoting configural processing (Rosa Salva et al., 2014). Thus, differences in visual strategies between different hymenopteran species for specific stimuli may depend upon a variety of factors that remain to be characterized. As both species shared a similar visual system (compound eyes and brain structure) due to their phylogenetic common history, it could be speculated that the difference in the use of holistic processing may be dependent of ecological differences, for example, in foraging (prey for wasps; flowers for honeybees) either through evolutionary adaptation or individual experience. Despite this difference for parameterized stimuli, we did observe some evidence that both species, despite their miniature brain, can holistically process visual information. This result suggests therefore that configural processing could be a more widespread visual solution in nature, and it would thus be of value to explore such a capacity in a wider range of vision-dependent species to understand how environmental and neurobiological contexts may influence visual recognition strategies.

The fact that two hymenopteran species show some evidence of holistic-like processing of complex visual stimuli leads to the interesting question of where in the insect brain such a process may take place. We hypothesize that mushroom bodies, sharing analogies with the higher cortical centers of vertebrate brains (Farris, 2008) and believed to be strongly linked to learning and memory processed in arthropod brains (Hammer and Menzel, 1995; Mizunami et al., 1998; Strausfeld et al., 1998; Hourcade et al., 2010; Devaud et al., 2015), should be the first structures to test for their implication in configural processing. In addition, Hymenopteran species such as bees and wasps do possess particularly developed mushroom bodies in comparison to other insects (Farris, 2008). For instance, the calyces of the mushroom bodies are doubled and expanded while receiving novel afferences from the visual part of the brain in comparison to *Drosophila* mushroom bodies (Farris, 2008; Avarguès-Weber and Giurfa, 2013). As the evolutionary development of mushroom bodies started back with ancestral parasitoid wasps (Farris and Schulmeister, 2011) that shared with bees spatial, visual, or olfactory learning need, the mushroom bodies are consequently considered as promoting learning abilities and flexibility (Giurfa, 2003; Chittka and Niven, 2009).

Finally, our new findings fit with the framework proposed by Chittka and Niven (Chittka and Niven, 2009) that large brains may not be necessary for processing seemingly complex stimuli, like faces, but rather the ecological conditions may enable the capacity to develop a brain that can use sophisticated strategies (Chittka and Niven, 2009; Chittka and Jensen, 2011; Chittka and Dyer, 2012). It is nevertheless likely that this new work has just scratched the surface of how hymenopteran insects, or even other animals may use configural processing, and it will be necessary to explore the very wide range of approaches applied in human psychophysics to build a more comprehensive understanding of these phenomenon in nature and in particular, how the impressive abilities of biological brains are possible, and what might be solutions that could be applied to machine vision (Kleyko et al., 2015; Cyr et al., 2017).

## Ethics Statement

Our research involves honey bees and wasps that are not animal models for which approval of an ethical committee is required. A minimum number of animals were used to resolve our scientific question. The animals remained free during the whole experiment and were not harmed in our experimental procedure.

## Author Contributions

AA-W and AD conceived the study and designed the experiments. Dd’A, MM, VF, DB, and AD performed the experiments. AA-W analyzed the data. AA-W and AD wrote the manuscript.

## Conflict of Interest Statement

The authors declare that the research was conducted in the absence of any commercial or financial relationships that could be construed as a potential conflict of interest.
